# Nanostructure Determines the Wettability of Gold Surfaces by Ionic Liquid Ultrathin Films

**DOI:** 10.3389/fchem.2021.619432

**Published:** 2021-02-05

**Authors:** Francesca Borghi, Matteo Mirigliano, Cristina Lenardi, Paolo Milani, Alessandro Podestà

**Affiliations:** CIMaINa and Dipartimento di Fisica “Aldo Pontremoli”, Università degli Studi di Milano, Milano, Italy

**Keywords:** ionic liquid, wettability, solid-like structure, gold, cluster-assembled film

## Abstract

Ionic liquids are employed in energy storage/harvesting devices, in catalysis and biomedical technologies, due to their tunable bulk and interfacial properties. In particular, the wettability and the structuring of the ionic liquids at the interface are of paramount importance for all those applications exploiting ionic liquids tribological properties, their double layer organization at electrified interfaces, and interfacial chemical reactions. Here we report an experimental investigation of the wettability and organization at the interface of an imidazolium-based ionic liquid ([Bmim][NTf2]) and gold surfaces, that are widely used as electrodes in energy devices, electronics, fluidics. In particular, we investigated the role of the nanostructure on the resulting interfacial interactions between [Bmim][NTf2] and atom-assembled or cluster-assembled gold thin films. Our results highlight the presence of the solid-like structured ionic liquid domains extending several tens of nanometres far from the gold interfaces, and characterized by different lateral extension, according to the wettability of the gold nanostructures by the IL liquid-phase.

## Introduction

Ionic liquids (ILs) are salts liquid at room temperature that are attracting a great interest in many disciplines for their low vapor pressure, good conductivity, wide electrochemical window and high thermal stability (Seddon, 1997). By accurately selecting the chemical composition of the anion and cation, which can be combined in a multitude of possibilities, the physico-chemical properties of the resulting bulk ILs can be tailored according to a specific task and application (Rogers, 2003).

ILs are largely employed as electrolytes in electrochemical and energy storage/harvesting devices (Ohno, 2011), as advanced lubricants (Zhou et al., 2009), as well as solvents in the chemical industry (Plechkova and Seddon, 2008). The permeability of the host matrix by the IL and the interactions taking place at the interface with the solid surfaces determine the suitability of the IL to a specific task. Nanostructured and porous materials have been employed in order to increase the interfacial area with the ILs, where the interactions occur (Vatamanu et al., 2013). In particular, nanostructured materials deposited in gas phase, as in the case of cluster-assembled thin films produced by Supersonic Cluster Beam Deposition (SCBD) (Wegner et al., 2006), have been proved to be easily incorporated into microtechnologies and appealing for the fabrication of electrochemical and energy devices thanks to their high and open porosity (Borghi et al., 2019a), that is easily wet and accessible by the ILs (Bettini et al., 2013; Soavi et al., 2016; Santaniello et al., 2018).

Ionic liquid wettability is of paramount importance since it controls the effective electrolyte permeability into the host materials, as in the case of energy storage/harvesting devices (Fedorov and Kornyshev, 2014), the performance of catalytic reactions at the interfaces (Steinrück and Wasserscheid, 2015), and it regulates the friction in the case in which ILs are used as lubricants (Fajardo et al., 2015). The main features that determine ILs wettability, related to their chemical composition and to the properties of the solid surfaces, are barely studied. The cation interactions with the solid surface, in respect of its structure and functionalized group (Chang et al., 2017; Bordes et al., 2018), the anion nature (Pereira et al., 2015), the stoichiometry of the solid surface (Bhardwaj et al., 2020) and its polarity (Pereira et al., 2015), as also water content in the IL (Sedev, 2011; Delcheva et al., 2015) are just a few among the features that determine ILs wettability of a surface, and in particular the contact angle which derives from the minimization of the free energy connected to the interfacial tensions of the three interfaces: solid-vapor, solid-liquid, and liquid-vapor (Plechkova and Seddon, 2008).

For many electrochemical and energy devices a small amount of IL is deposited on surfaces which are usually rough and chemically heterogeneous; in these systems, the Young equation cannot be used to describe the contact angle at the interface (Plechkova and Seddon, 2008). For rough and defects-rich surfaces, the base of the droplet may deviate from a circular shape causing the presence of local contact angles corresponding to local minima of the free energy of the system.

In addition to this non-ideal conditions, the use of a small amount of IL and the corresponding reduction of droplets size, enhance the contribution derived from the line tension free energy (Rowlinson and Widom, 1989): once the droplet size reaches the sub-micron dimensions, line tension effects can no longer be neglected (Swain and Lipowsky, 1998), and the size dependence of the wetting behavior for such nano-droplets is described by a modified Young’s equation (Delcheva et al., 2015).

The presence of nanometers thick (ultrathin) ionic liquid films surrounding large droplets of IL deposited on smooth mica surface (Liu et al., 2006; Beattie et al., 2013) has been reported. The origin of these ultrathin films have not been unambiguously determined, whether they were originated from the submicron-sized IL droplets spreading on the interface, or if the IL films have been formed upon evaporation of the solvent and affected by pinning phenomena on the surface (Beattie et al., 2013; Delcheva et al., 2015; Sheehan et al., 2016).

In this work we focused on the characterization of a tiny amount (few µl) of an imidazolium-based ionic liquid ([Bmim][NTf2]) deposited on gold nanostructured surfaces that are commonly employed as conductive paths and electrodes in devices. In particular, the IL wettability at the nano- and meso-scale has been evaluated on gold thin films characterized by different structural properties. In fact, the elemental building blocks (atoms deposited by thermal evaporation or clusters deposited by SCBD) that constitute the gold thin films provide different structural properties to the layers (Mirigliano et al., 2019; Mirigliano et al., 2020). Interestingly, the formation of extended solid-like domains of ionic liquids, similar to those observed in Refs (Bovio et al., 2009; Galluzzi et al., 2018a; Borghi et al., 2019b, Borghi et al., 2021; Borghi and Podestà, 2020), were demonstrated, with more relevance on the evaporated gold thin film, where the liquid phase wets less the surface.

## Materials and Methods

### Deposition of Gold Thin Films and ILs

Nanostructured gold have been deposited on oxidized silicon substrates by thermal evaporator (Edwards, E306A model) in vacuum for atom-based layers and by Supersonic Cluster Beam Deposition (SCBD) (Wegner et al., 2006) in the case of cluster-assembled thin films.

SCBD apparatus schematically consists of three differentially pumped vacuum chambers: Au clusters are produced in gas phase through the ablation of a gold target rod by a pulsed discharge plasma ignited during the injection of a high pressure inert gas in the Pulsed Microplasma Cluster Source (PMCS) (Wegner et al., 2006). The gold clusters and the inert carrier gas mixture is expanded into vacuum to form a seeded supersonic beam that impinges on the substrate fixed on a sample holder perpendicular to the beam (Mirigliano et al., 2020). The mass deposited is monitored by a quartz microbalance integrated in the sample holder.

A thin stencil mask has been introduced for both techniques between the substrates and the gold particles beams, in order to deposit patterned nanostructured gold thin films characterized by the same macroscopic geometrical features. In particular, the gold tracks are 1 mm large and 7 mm long.

1-Butyl-3-methylimidazolium bis(trifluoromethylsulfonyl)imide ([Bmim][NTf2]) has been kept in an ultrahigh vacuum chamber (10^−6^ mbar) for several days before the experiments, in order to reduce water contamination. Methanol (purity 99.8%, HPLC, from Fluka) was distilled twice, in order to decrease the amount of non-volatile contaminants as well as the water content. We performed the drop-casting deposition of 10 μL droplet of (1:1000) [Bmim][NTf2]/methanol diluted solution on both the gold substrates.

### Characterization of the Interfaces at the Nanoscale

The investigation of the morphological and mechanical properties of the IL/gold interfaces was performed by a Multimode 8 AFM (Bruker), by exploiting different modes depending on whether the measurements were carried out in air or in liquid environment.

The morphology of the nanostructured gold thin films and the ionic liquid features exposed to the air have been characterized by AFM in tapping mode, while force vs. distance/indentation curves were done according to a point&shoot strategy (Borghi et al., 2019b) by using rigid tapping mode cantilevers; in particular, NCHV probes from Bruker, with resonance frequency around 300 kHz, force constant k = 40 N/m, and nominal tip radius 8 nm, were used for these experiments.

The interface between gold thin films and the ionic liquid, in a complete wetting condition, was characterized in Peak-Force Tapping Mode, using silicon nitride cantilevers mounting single crystal silicon tips, with nominal radius 12–30 nm, resonance frequency in the range 100–200 kHz, and force constant k = 0.7 N/m.

The topographic maps have been recorded with a sampling resolution of 1–5 nm/pixel using a scan rate of approximately 1 Hz. From flattened AFM images, the root-mean-square surface roughness Rq was calculated as the standard deviation of surface heights (Podesta et al., 2015).

Force-distance curves were recorded along a grid spanning a region of a topographic map previously acquired by AFM imaging in tapping mode. The nano-mechanical analysis was then performed by fitting the Hertz model (Butt et al., 2005) to the experimental force vs. indentation curves (F_Hertz_ = $\frac{4}{3}{\text{E}}^{\text{*}}\sqrt{\text{R}}$ ${\text{δ}}^{\frac{3}{2}}$), where δ represents the indentation, E* is the effective Young’s modulus (including Poisson’s ratio), and R is the tip radius. The Hertz model is a good approximation of the elastic response of the solid-like IL layers because the total indentation is not large compared to both the probe radius and the IL layers thickness (Galluzzi et al., 2018a). In force vs. indentation curves, penetration events observed at high forces are called breakthrough events.

### Optical Characterization

Optical images of the IL/gold interfaces were acquired using a Zeiss Axio imager A1 microscope. We have collected images at ×20 magnification, in order to have a resolution sampling of approximately 366 nm/pixels, and images at ×4 magnification in order to have a wide field of view.

The estimation of the geometrical properties of the objects identified as IL droplets on the gold surface by optical investigation, has been carried out by converting optical images into grayscale maps, and by defining a gray threshold for the binarization of the image. According to the method explained in Refs (Borghi et al., 2018; Mirigliano et al., 2019), a thresholding method was chosen to perform a segmentation of the images: all the values below the chosen level are approximated with 0 (the gold substrate), while the others with 1 (ionic liquid droplets).

The evaluation of the perimeter *p*, the 2D-projected area *a* and the circularity *c* of the droplets (*c* = 4aπ/$p^{2}$) was performed in Matlab environment.

### X-ray Photoelectron Spectroscopy (XPS)

XPS experiments were performed at room temperature under UHV condition with a PHI 5600, equipped with a hemispherical electron analyzer and a monochromatized X-ray source (Al Kα = 1486.6 eV, ΔE = 0.48 eV). The high-resolution spectra were acquired in constant step energy mode with Epass = 23.8 eV. The overall energy resolution was 0.8 eV; while the pressure in the experimental chamber during experiments was lower than 4 × 10^−9^ mbar. The binding energy scale was calibrated via the Au 4f7/2 core level line (located at 84.00 eV) of a clean polycrystalline Au sample. Voigt line-shape and Shirley background were used to fit the peaks and the background, accordingly. Least square curve-fitting was performed using the programs KolXPD and Igor Pro.

## Results and Discussion

### Structural Properties of Gold Thin Films

The dimensions and the organization of the gold nanoparticles, which depend on the particle kinetic energy at the impact with the surface, the particles diffusion and coalescence processes, determine the structure of the resulting thin films (Milani and Iannotta, 1999; Jensen, 1999), as well as their porous structure and interfacial morphology (Konstandopoulos, 2000). Gold clusters, deposited by SCDB, are characterized by a bimodal distribution of their size effective diameters, with very small aggregates peaked at 0.40 nm, and a population of largest clusters around 6 nm, as reported in Ref. Mirigliano et al. (2019).

**FIGURE 1:**
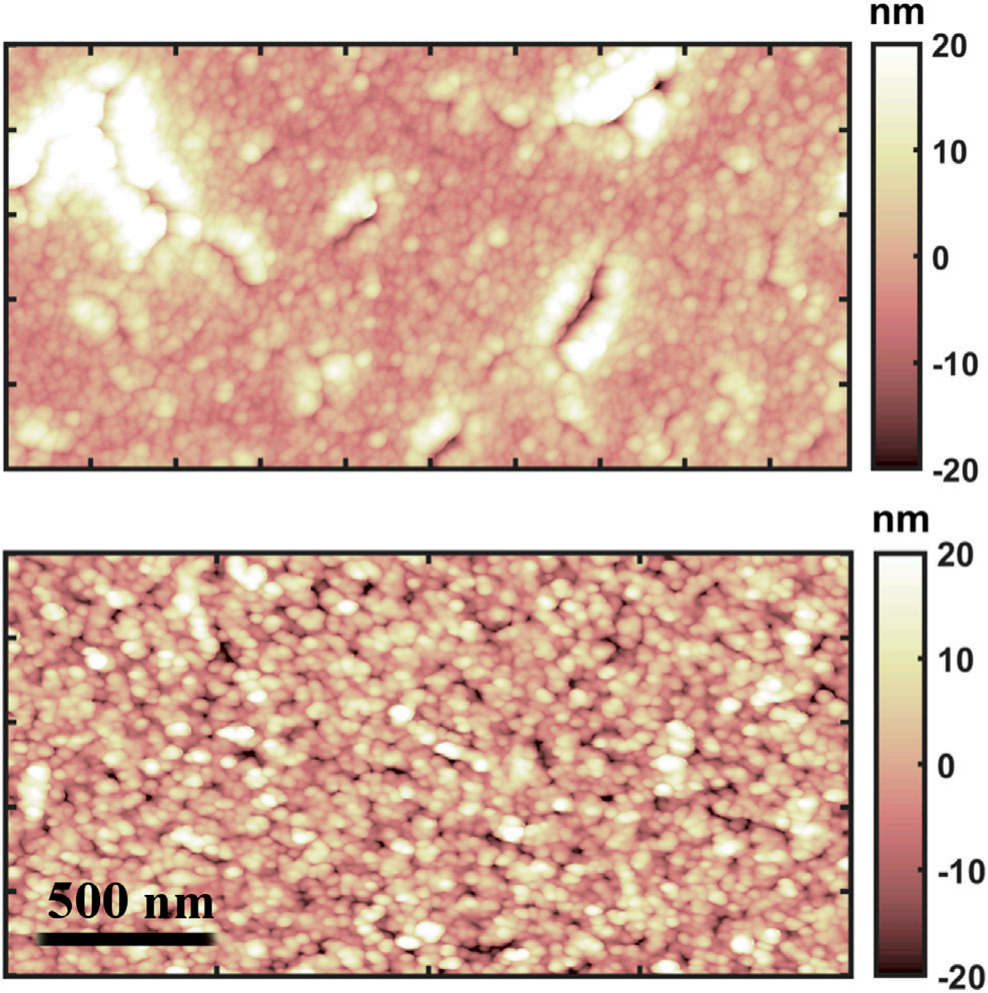
AFM morphological maps of the evaporated (up) and cluster-assembled (down) gold thin films.

In Figure 1, typical morphological maps of the two differently deposited gold thin films are shown: despite their similar surface roughness, reported in Table 1, hundreds of nanometer wide cracks appear on the evaporated thin film, while the cluster-assembled thin film appears more homogeneous.

**TABLE 1:** Morphological properties of the evaporated and cluster-assembled gold thin films.

Sample	Roughness (nm)	Thickness (nm)
Evaporated gold	8.5 ± 1.6	180 ± 5
Cluster-assembled gold	9.8 ± 2.0	32 ± 2

The nanogranular structure of the cluster-assembled thin films introduces a huge number of defects and junctions affecting also the electrical transport and causing a non-linear behavior (Mirigliano et al., 2019; Mirigliano et al., 2020). The structural properties and the remarkable presence of surface defects, which affect the electrical properties of the gold cluster-assembled thin films, may also modify the local electrostatic properties of the surface (Yajadda et al., 2011) and hence the local wettability of the ionic liquid on the surface, as discussed in Ref. Pereira et al. (2015).

**FIGURE 2:**
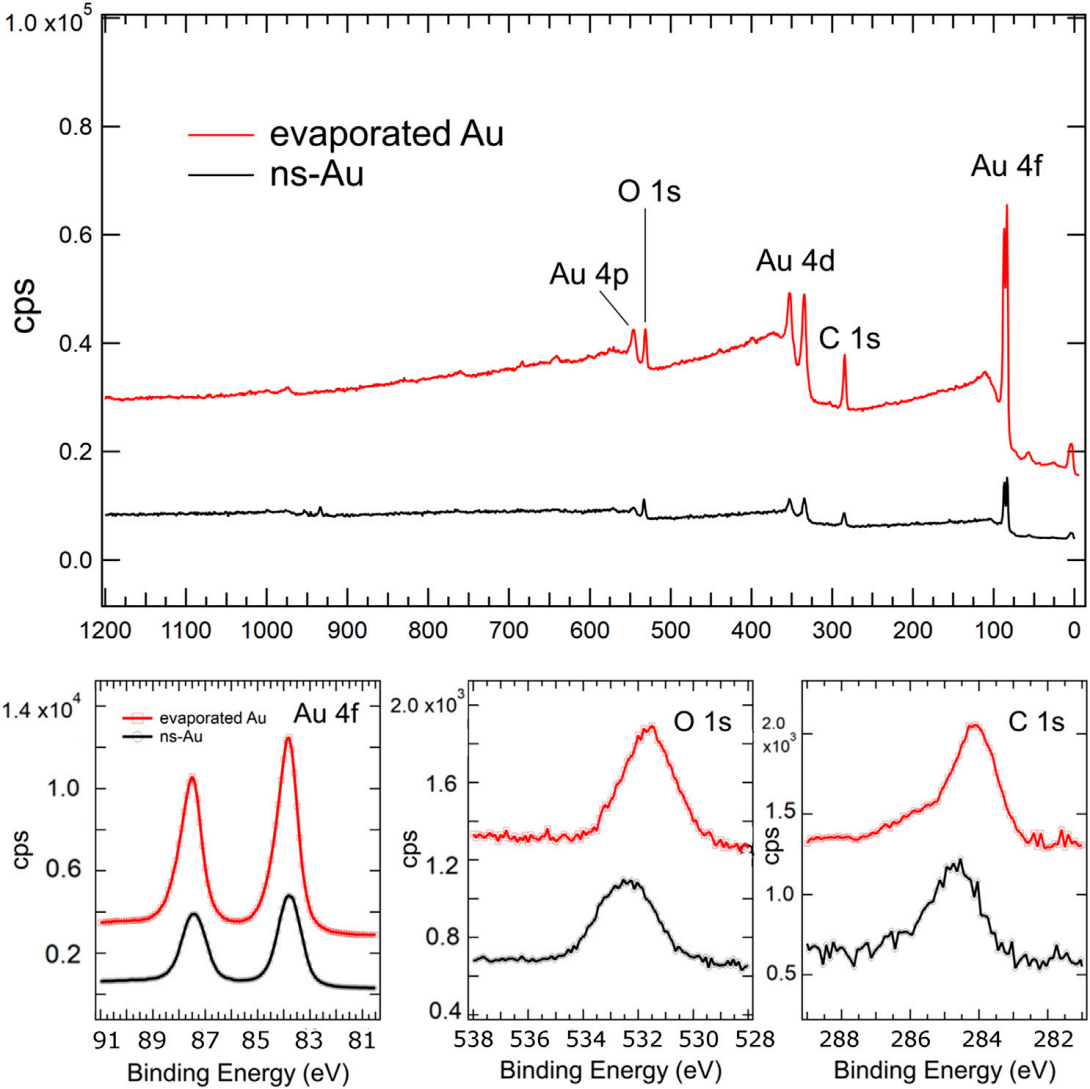
High-resolution XPS scans acquired on evaporated and cluster-assembled gold thin films; the wide spectra (up) and the peaks revealing Au4f, O1s and C1s (below) are shown.

In order to characterize the chemical composition of the surfaces, we have performed an X-ray photo-electron spectroscopy (XPS) of the two pristine samples. The wide spectra and the regions of the Au 4f, O1s and C1s peaks have been shown in Figure 2.

The chemical composition and stoichiometry of the surface strongly influence the surface wettability of a liquid. In Ref. Bhardwaj et al. (2020). the authors highlight an increase in the contact angle of an imidazolium-based ionic liquid with increasing oxygen/carbon ratios of boron-doped nanocrystalline diamond (BDD) thin-film electrodes, as determined by XPS spectra. They suggest that the alkyl side chain of the imidazolium ring of the cation might interact more strongly with the hydrogen rich surface due to hydrogen-bonding interactions, while the increased surface oxygen coverage, may orient the alkyl side chain away from the surface toward the bulk of the liquid (Bhardwaj et al., 2020). In our gold thin films the O/C ratio calculated by XPS spectra is quite similar: it is 0.4 for the evaporated and 0.5 for cluster-assembled gold thin films. No other features characterize the two different gold samples significantly, as it is highlighted by the wide spectra.

### [Bmim][NTf2] – Methanol Binary Mixture Deposited on Gold Films

In order to obtain a low surface coverage of the ionic liquid, we have drop-casted 10 µL of a 1:1000 [Bmim][NTf2]/methanol diluted solution onto the gold surfaces, waiting for the complete evaporation of the volatile solvent. The dynamics implied in the solvent evaporation process could be complex (Deegan et al., 1997) and it could have a relevant role in the resulting geometrical and structural properties of the ionic liquid droplets formed on the substrate.

The evaporation of the solvent induces cooling and temperature gradients into the droplets, which may promote a corresponding surface tension gradients, with resulting Marangoni effect (Scriven and Sternling, 1960). The picture is complicated by the fact that during the evaporation the solvent, as soon as the volume of the drop decreases, the concentration of the ionic liquid increases; many binary mixture properties, as the excess molar volume (Geppert-Rybczyńska et al., 2014), the IL surface tension and the resulting IL fluxes inside the drop, can be modified. In Ref. Andreatta et al. (2014). detailed experimental results showing the changes in surface tensions of [Bmim][NTf2]/methanol mixtures, as a function of the alkyl chain length of the ionic liquid and of the temperature are shown. Although we could not find in literature a study concerning the phase diagram of a binary mixture composed by methanol and [Bmim][NTf2], interesting works regarding the phase diagram of imidazolium-based ionic liquid and methanol (Shimomura et al., 2010; Vale et al., 2011), as also [Bmim][NTf2] and other alcohol mixtures (Crosthwaite et al., 2004; Pereiro et al., 2010; Domańska et al., 2012), suggest that at room temperature the miscibility of the [Bmim][NTf2] into methanol can dramatically be affected by the relative concentration of the two liquids.

**FIGURE 3:**
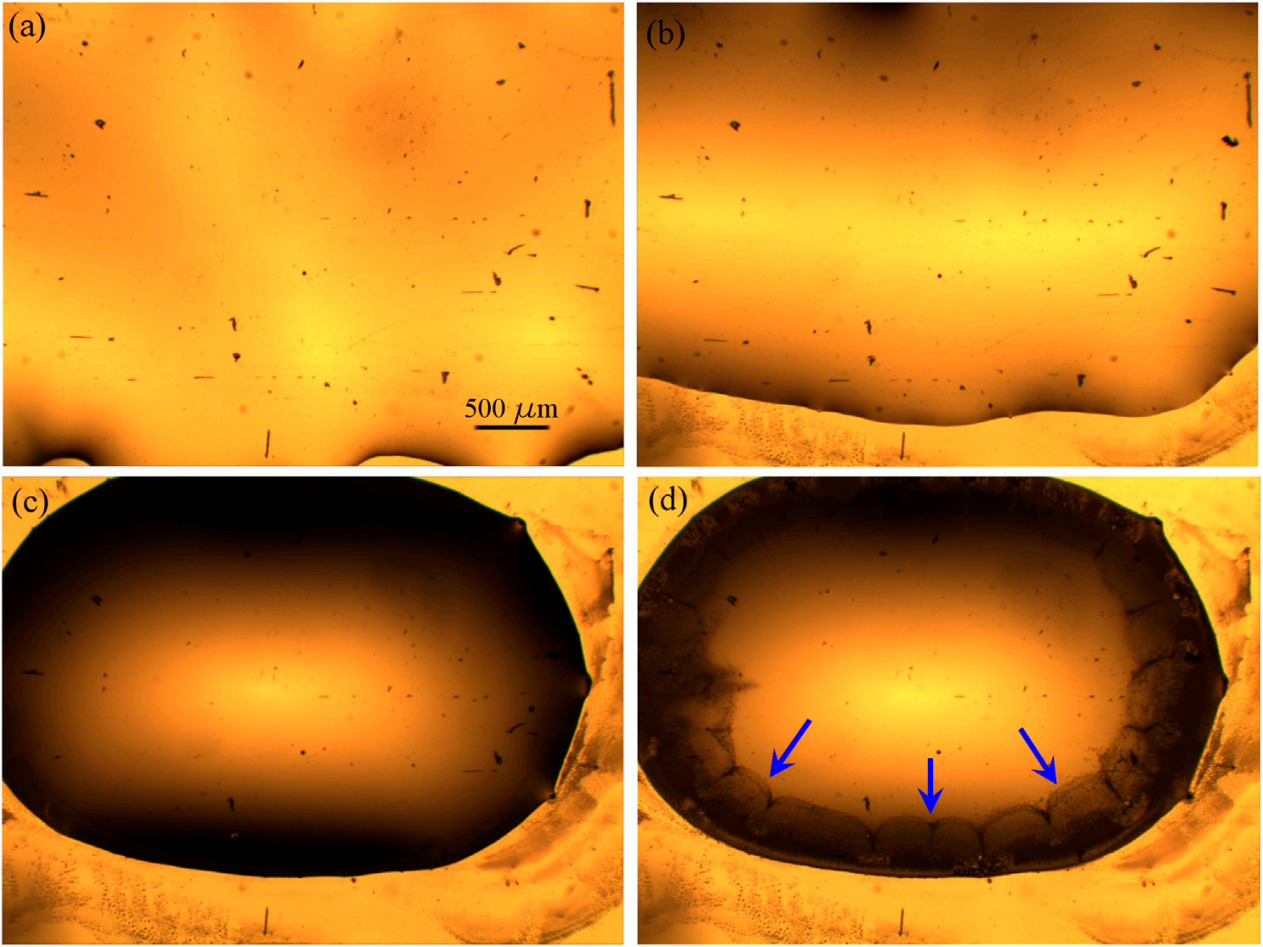
Optical images of 10 µL of a [1:1000] diluted [Bmim][NTf2]/methanol solution deposited on evaporated gold thin film (×4 magnification), at successive times (Δt = 15 s) from **(A–D)**.

In our experiments, after the drop-casting of [Bmim][NTf2]/methanol solution, the binary mixture rapidly spreads on the entire gold surface and after few seconds it starts to shrink into a more compact big drop, driven by the evaporation process of the solvent. With a slower kinetics, the evaporation of the methanol continues from the upper interface of the [Bmim][NTf2]/methanol big drop until a partial segregation of the IL starts on the edge of the drop, because of the local increase of the relative concentration of IL/methanol. The IL drops dispersed into methanol diffuse until their sedimentation on the gold surface, where the drops change their shape from a spherical into a hemispherical one, characterized by irregular perimeters. In Figure 3, snapshots acquired few seconds after the deposition of 10 µL of IL/methanol solution on the evaporated gold thin film, show a sequence of the methanol evaporation process. The black features represent impurities and micrometer defects on the gold surface.

**FIGURE 4:**
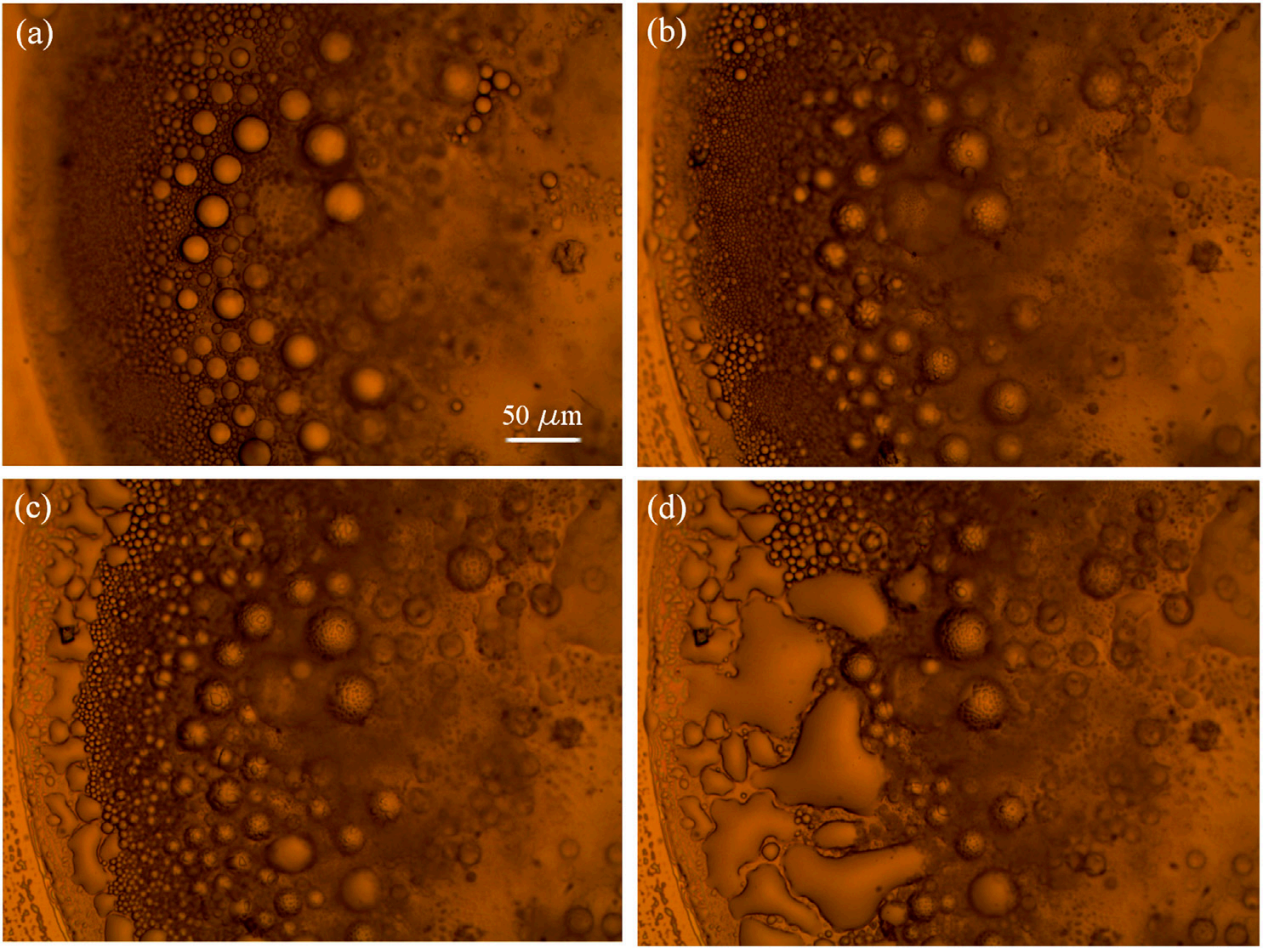
[Bmim][NTf2] droplets dispersed in methanol at the edge of a big drop on gold cluster-assembled surface, at successive times.

Immediately after the drop-casting of the methanol/IL solution, the drop spreads over the entire gold surface and after few seconds it begins to reduce the contact area with the substrate (Figures 3A,B) and to collapse into a more compact drop (Figure 3C). After few minutes, because of the evaporation of the methanol from the surface of the entire drop, and in particular because of the increase of the relative concentration of IL/methanol on the edge of the drop, the separation of ionic liquid from the methanol phase promotes the formation of IL droplets close to the contact line (indicated by the blue arrows in Figure 3D), as it is expected (Deegan et al., 1997). In Figure 4, snapshots which exhibit the IL droplets disperse in methanol, formed at the edge of the IL/methanol bigger drop on nanostructured cluster-assembled surface, are shown. The kinetics of the IL droplets sedimentation on the gold film is slower than the initial evaporation of methanol, since it takes approximately 30 min for the IL droplets dispersed in methanol (Figure 4A) to reach and attach to the gold surface (Figure 4D).

Similar behavior of the main IL/methanol drop has been recorded on both the gold surfaces, although the final wettability of the IL on the gold surface depends on the specific gold nanostructure, as it is discussed below.

### [Bmim][NTf2] Wettability of the Gold Thin Films at the Macroscale

**FIGURE 5:**
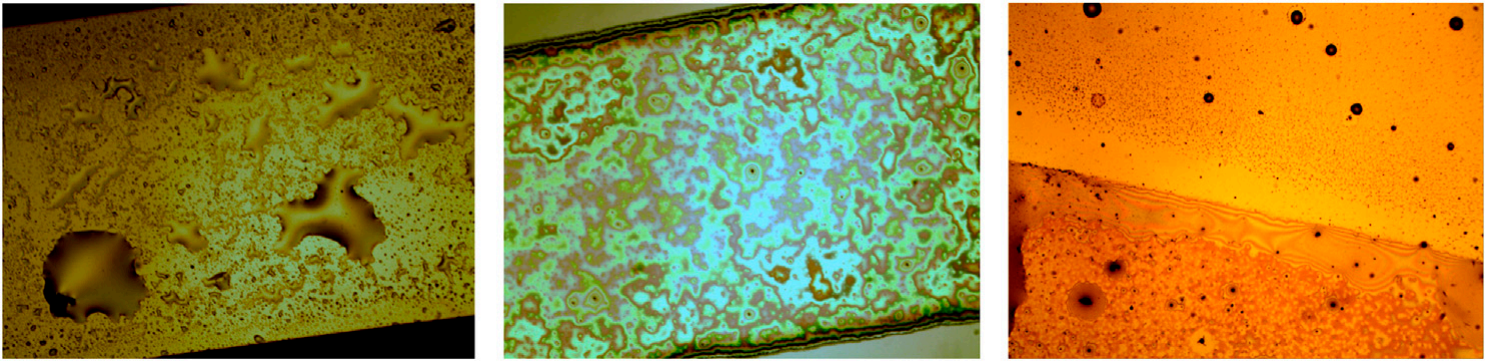
Optical images of the 1 mm wide path of evaporated gold **(A)**, cluster-assembled gold thin film **(B)** and border region of the two films **(C)**, covered by a small amount of [Bmim][NTf2] deposited by drop casting 10 µL of 1:1000 [Bmim][NTf2]/methanol diluted solution. The scale bar is the same for all the images.

The overall arrangement of the small amount of ionic liquid after the complete evaporation of methanol on the gold surfaces reveals a different wettability depending on the nanostructure: large compact droplets pinned at the macroscopic defects on the evaporated atom-assembled thin films (Figure 5A), and a thin ionic liquid wetting layer on the nanostructured cluster-assembled gold sample (Figure 5B).

In Figure 5C a border region between the evaporated and the cluster-assembled thin film deposited on it, covered by ionic liquid, is shown: the IL remains confined onto the nanostructured cluster-assembled thin film, without overflowing out of it, despite the continuity of the gold border region.

**FIGURE 6:**
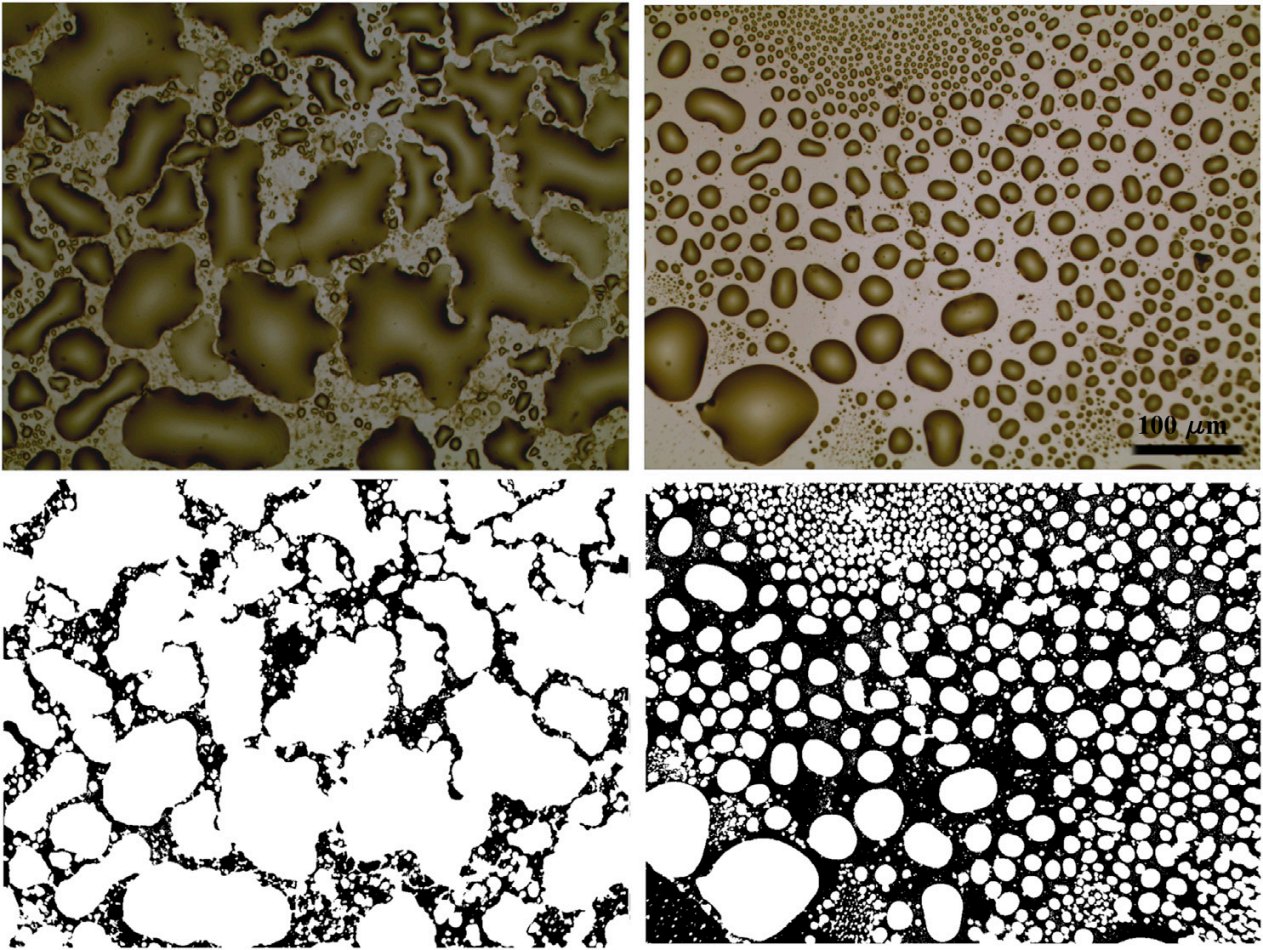
Optical images (×20 magnification) acquired on cluster-assembled gold thin film **(left)** and on evaporated gold **(right)** covered by 3 µL of 1:1000 diluted [Bmim][NTf2]/methanol solution, and the corresponding binarized images below. The scale bar is the same for all the images.

More detailed pictures of the gold samples covered by a smaller amount of IL, obtained by depositing 3 µL of [Bmim][NTf2]/methanol solution, have been acquired in order to analyze the geometrical features characterizing the IL droplets on the surface. Two exemplifying images acquired on samples at high magnification are shown in Figure 6.

**FIGURE 7:**
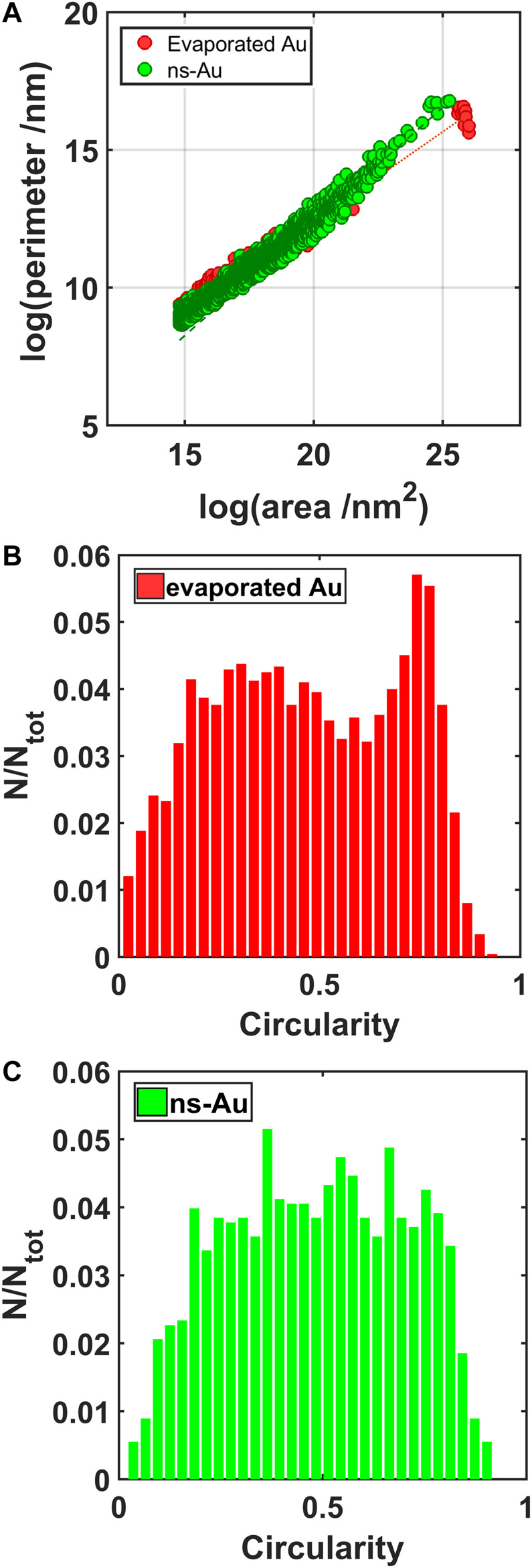
Perimeter vs. area of the IL droplets in log-log scale **(A)**; circularity of the IL droplets formed on the evaporated gold **(B)** and on the cluster-assembled gold thin film **(C)**.

By analyzing the geometrical properties of the objects identified in the binarized images, it is possible to recognize different interactions taking plase between the ionic liquid and the gold thin films. The relation between the perimeters and the areas of the IL droplets stresses the irregularity at different scales (fractal dimension) (Kirkby, 1983) of the objects analyzed. In particular, the angular coefficient α of the perimeter vs area relation characterizing the largest objects, in log-log scale (Figure 7A), is 0.65 for the evaporated and 0.83 for the cluster-assembled thin film; the former is similar to the case of a perfect rounded objects (*α* = 0.5), while it is more fractal for the latter. The irregularity of the contact line of the droplets on the gold nanostructure can be attributed to the clusters defects and asperities which act as pinning centers.

This irregularity is highlighted also by the distribution of the circularity values (Figures 7B,C), obtained by the geometrical properties of the droplets on the two different gold surfaces: the plots show a broad distribution corresponding to the cluster-assembled film, while a peak in the circularity of the droplets formed on the evaporated gold around 0.8 highlights a more regular and circular geometry.

The total surface coverage due to the ionic liquid on the evaporated gold surface is 60%, while it increases up to 80% for the cluster-assembled thin film. More compact and rounded shape characterizes the IL droplets on the evaporated gold film.

These geometrical features emphasize what it was qualitatively evident on Figure 5: the nanostructured cluster-assembled material shows better wettability by [Bmim][NTf2]. Both the structural properties of gold clusters, such as surface defects which can act as pinning sites for ionic liquids, and their electrical properties, which contribute to a more polar surface compared to atom-based gold thin film, can improve the local wettability of the ionic liquid, as also suggested by other works concerning similar systems (Beattie et al., 2013; Delcheva et al., 2015; Pereira et al., 2015).

### Solid-like Ionic Liquid Structures

The formation of solid-like ionic liquid terraces on flat (mica, amorphous silica, and oxidized silicon) (Bovio et al., 2009; Galluzzi et al., 2018a) and nanostructured thin films (Borghi et al., 2019b; Borghi et al., 2021) has been extensively reported in literature. IL thin film patches and droplets coexist on the surface, in condition of surface confinement, as described in Ref. Borghi and Podestà (2020). Here, the term solid-like refers to changes of many structural and functional properties of the ionic liquid which form multi-layered micrometer-wide terraces, characterized by a 0.6 nm (Bovio et al., 2009; Ballone et al., 2012) fundamental step, which extend from the surface up to several tens or even hundreds of nanometers (Bovio et al., 2009; Galluzzi et al., 2018b; Borghi et al., 2019b; Borghi et al., 2021). These structures are rigid, characterized by an apparent Young Modulus of the order of 1 GPa (Galluzzi et al., 2018a; Borghi et al., 2019b; Borghi et al., 2021), and possess electrical insulating character (Galluzzi et al., 2018b).

Recent works (Comtet et al., 2017; Lainé et al., 2020) describe phase transition of confined IL between conductive interfaces into a glassy-like state. This behavior is interpreted in terms of a freezing transition shift, which is related to the surface energy of the liquid with respect to the confining surface. If the wetting of the walls matrix by the ionic liquid in its solid-phase is favored compared to the wetting by the ionic liquid in the liquid-phase, the phase transition in confining conditions may occur at highest temperature than in bulk conditions. As it has been demonstrated for nanostructured oxidized silicon surface (Borghi et al., 2019b) and nanostructured carbon surface (Borghi et al., 2021), the nanostructure does not prevent the formation of ordered ionic liquid structures, which maintain their solid-like behavior.

**FIGURE 8:**
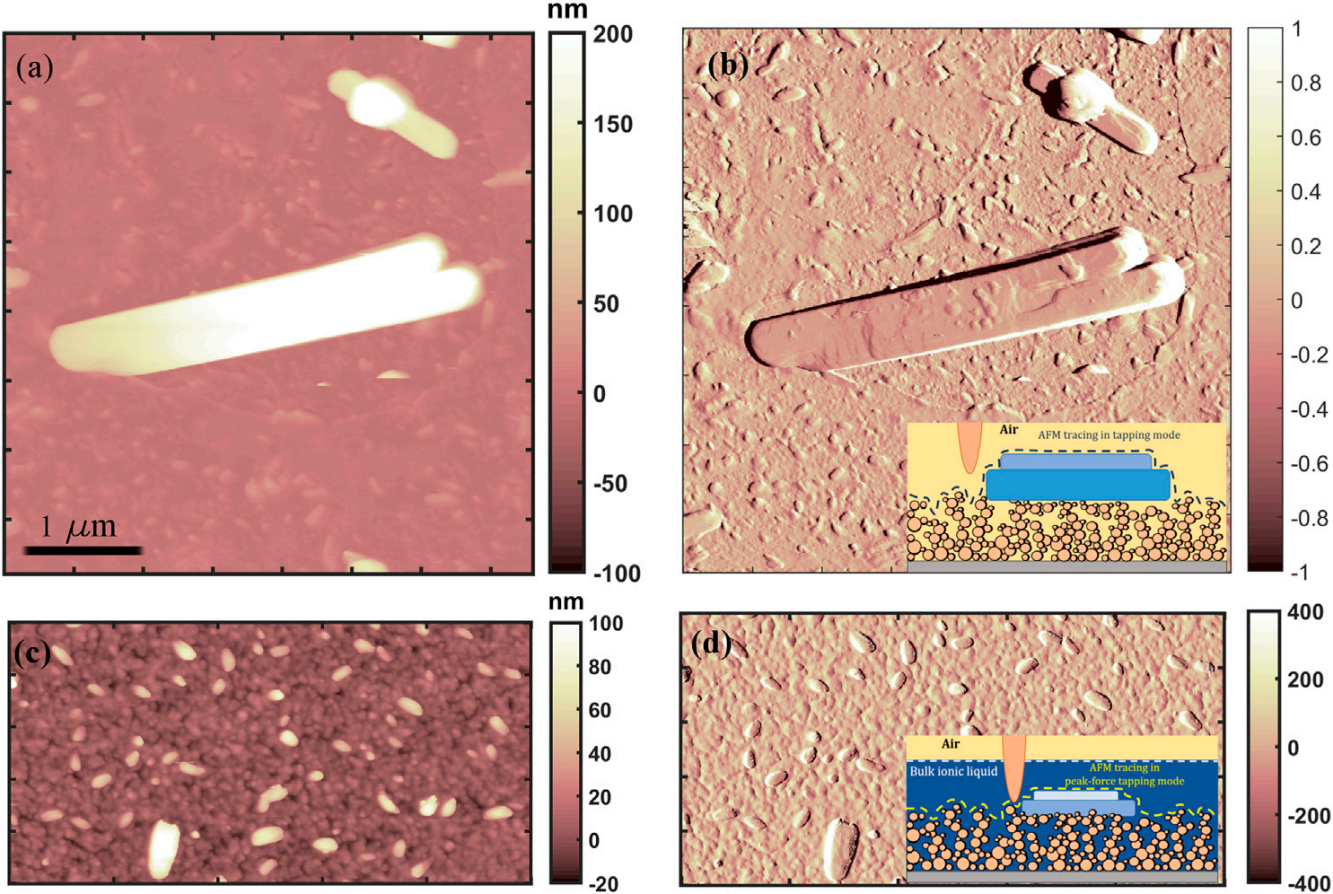
Representative AFM morphological **(left)** and amplitude error **(right)** maps of structured [Bmim][NTf2] formed on evaporated gold **(A,B)** and on cluster-assembled **(C,D)** thin films.

Our results show the existence of highly ordered ionic liquid structures, several micrometer large and hundreds of nm high, on evaporated gold surface (Figure 8, top), as well as the presence of smaller ordered ILs domains on gold cluster-assembled thin film (Figure 8, bottom). The structured ionic liquid domains formed on the evaporated gold thin film are one order of magnitude larger (approximately few µm^2^) than the small objects grown on the cluster-assembled thin film, they are exposed to air (as it is shown in the inset of Figure 8B) and not covered by a thin film of ionic liquid in liquid phase, as the small objects formed on cluster-assembled film.

The ionic liquid structured in ordered geometry on evaporated atom-based gold films have sharp and well-defined borders, while the ones on nanostructured cluster-assembled thin film are more rounded; as discussed before, the surface with lowest wettability by the liquid phase of the IL form more stable solid-like features, whose mechanical properties will be discussed here after.

By assuming that the surface energies of evaporated gold/air and of ns-Au/air are comparable, the results obtained on the gold surfaces reinforce the hypothesis that the IL transition to a solid-like phase is promoted by a higher surface energy of the liquid phase compared to the solid one, as also demonstrated in Refs Comtet et al. (2017) and Lainé et al. (2020), which is associated to a lower wettability of the liquid phase on the confining surface (Evans, 1990).

The larger and well-ordered boundary geometry of the structured ionic liquid formed on the evaporated gold is probably promoted by the more compact morphology and electrically conductive character (Mirigliano et al., 2019) of the gold evaporated surface, while the smaller and more rounded objects grown on the nanostructured cluster-assembled film can be promoted by the presence of an extremely large number of surface defects, that also affects the electrical properties of the gold cluster-assembled thin films (Yajadda et al., 2011). These structural and functional properties of the cluster-assembled system may affect the overall wettability of the ionic liquid (by increasing it), as also the lateral extension of the resulting ordered solid-like ionic liquid (by reducing their dimensions).

**FIGURE 9:**
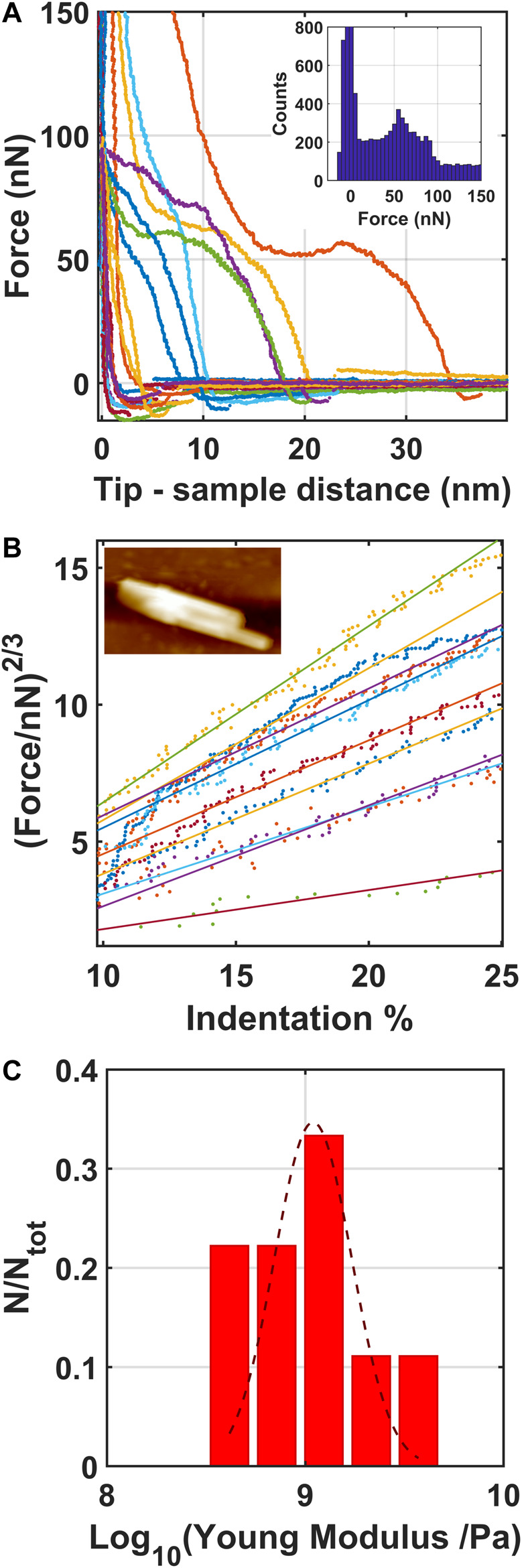
Force curves acquired on the structured [Bmim][NTf2] covering evaporated gold thin film **(A)**, the corresponding Hertzian fit (Butt et al., 2005) of the linearized FCs **(B)** and the corresponding distribution of the logarithmic values of the apparent Young Modulus calculated by the force-curves **(C)**. A Gaussian fit is superimposed to the experimental data. In the inset of figure a, the distributions of the forces are shown, while in the inset of figure b the morphological map of the structured object we have investigated by fcs has been reported.

Force vs. distance curves (FCs) have been acquired in order to analyze the indentation region of AFM tip into the structured ionic liquid formed on the evaporated gold thin film. Figure 9A report the force curves acquired on the structured object reported as inset in Figure 9B. The distribution of the forces, shown in the inset of Figure 9A, highlights the breakthrough forces corresponding to the rupture events of the structured ionic liquid domains by AFM tip. In particular they appear at 50 and 80 nN, in good agreement with the breakthrough forces of solid-like structured ionic liquid terraces formed on nanostructured oxidized silicon surface (Borghi et al., 2019b), analyzed with a AFM tip characterized by the same radius. Accordingly, the median value and the standard deviation of the apparent Young Modulus of the structured ionic liquid terraces (whose distribution is reported in Figure 9C), calculated by analysing the force indentation curves with the Hertz contact mechanical model (Butt et al., 2005), as shown in Figure 9B, is 1.1 ± 0.1 GPa, which confirms the solid character of the structured ionic liquid terraces formed on evaporated gold thin film. This is in good agreement also with the apparent Young Modulus of solid-like islands formed on flat (Galluzzi et al., 2018b) and nanostructured oxidized silicon (Borghi et al., 2019b) and nanostructured carbon (Borghi et al., 2021) substrates.

It has not been possible to test the mechanical properties of the smallest IL objects grown on the cluster-assembled gold film since their small dimensions. However, the corresponding AFM images (shown in Figure 8-down) have been acquired on a gold cluster-assembled sample completely covered by the ionic liquid (as it is schematically shown in the inset of Figure 8D): the ILs rounded objects of Figures 8C,D appear at the bottom of the liquid phase, at the interface with the gold nanostructure. The AFM images have been acquired by applying a force that corresponds to 5 nN in order to break the liquid on the surface (that is not reproduced in the image), while the IL features remained in the AFM image suggest a solid-like character, which should be confirmed in the future.

## Conclusion

This work experimentally demonstrates that the interactions occurring at the interface between a gold film and an ionic liquid are influenced by the different nanoscale morphologies of the conductive surface. In particular, the surface wettability and the structuring of [Bmim][NTf2] into solid-like terraces are modified according to the nature of the gold building blocks (atoms or clusters), which constitute the thin films and provide different structural and conductive properties to the film surface(Mirigliano et al., 2019).

Our results stress the role of the structural and electronic defects in cluster-assembled thin film in the resulting ionic liquid wettability and they also provide the evidences of the correlation between the ionic liquid wettability and the formation of the well-defined solid-like ionic liquid structures.

The formation of solid-like terraces is not hampered by the roughening of the surface and by its conductive nature; actually, atom-assembled gold thin films, showing standard ohmic conduction, induces also the formation of the largest and highest ionic liquid solid-like structures.

## Data Availability Statement

The raw data supporting the conclusions of this article will be made available by the authors, without undue reservation.

## Author Contributions

Conceptualisation: FB, AP; methodology – samples fabrication: FB, MM; methodology – XPS: CL; methodology – AFM and OPTICAL MICROSCOPY: FB; data analysis: FB; original draft writing: FB, PM, AP; draft reviewing and editing: FB, MM, PM, AP, CL; supervision: FB, PM, AP.

## Conflict of Interest

The authors declare that the research was conducted in the absence of any commercial or financial relationships that could be construed as a potential conflict of interest.
